# Predictors of lower limb fractures in general Japanese: NIPPON DATA90

**DOI:** 10.1371/journal.pone.0261716

**Published:** 2022-02-02

**Authors:** Yoshino Saito, Katsuyuki Miura, Hisatomi Arima, Takehito Hayakawa, Naoyuki Takashima, Yoshikuni Kita, Nagako Okuda, Akira Fujiyoshi, Toshiyuki Iwahori, Naoko Miyagawa, Keiko Kondo, Sayuki Torii, Aya Kadota, Takayoshi Ohkubo, Akira Okayama, Tomonori Okamura, Hirotsugu Ueshima, for the NIPPON DATA90 Research Group

**Affiliations:** 1 Department of Nursing Faculty of Health Science, Aino University, Osaka, Japan; 2 Department of Public Health, Shiga University of Medical Science, Shiga, Japan; 3 NCD Epidemiology Research Center Shiga University of Medical, Science, Shiga, Japan; 4 Department of Preventive Medicine and Public Health, Fukuoka University, Fukuoka, Japan; 5 Research Center for Social Studies of Health and Community Ritsumeikan University, Kyoto, Japan; 6 Department of Public Health, Faculty of Medicine, Kindai University, Osaka, Japan; 7 Tsuruga Nursing University, Fukui, Japan; 8 Department of Health Science, Kyoto Prefectural University, Kyoto, Japan; 9 Wakayama Medical University, Wakayama, Japan; 10 Department of Preventive Medicine and Public Health, Keio University School of Medicine, Tokyo, Japan; 11 Department of Hygiene and Public Health, Teikyo University School of Medicine, Tokyo, Japan; 12 Research Institute of Strategy for Prevention, Tokyo, Japan; Medical College of Wisconsin, UNITED STATES

## Abstract

**Objective:**

This study aimed to investigate the incidence rates and predictors of lower limb fractures in a general Japanese population.

**Methods:**

NIPPON DATA is a nationwide, long-term, prospective cohort study of individuals who participated in the National Cardiovascular Survey Japan and the National Nutrition Survey in 1990. Overall, 3,134 individuals (1,827 women, 1,307 men) who participated in follow-up assessments in 1995, 2000, and/or 2006 were included in the present analysis. The outcomes of this study were lower limb fractures (including proximal femur fractures).

**Results:**

The mean age at baseline was 63.8 years in women and 63.1 years in men. The average body mass index (BMI) was 23.3 kg/m^2^ in women and 22.9 kg/m^2^ in men. During a mean follow-up of 12.1 years, 271 total lower limb fractures were observed. In women, older age, lower BMI, and less intake of vegetables were associated with increased risks of proximal femur fractures. With regard to the outcome of total lower limb fractures, less intake of vegetables and regular exercise were significant predictors in women. Calcium intake was not significantly associated with proximal femur or total lower limb fractures. There were no significant predictors of proximal femur or total lower limb fractures in men, except for age.

**Conclusions:**

Aging was a significant risk factor for proximal femur and total lower limb fractures in both men and women. With regard to modifiable risk factors, low BMI and low intake of vegetables were associated with increased risks of proximal femur and/or total lower limb fractures in the general population of Japanese women.

## Introduction

Owing to the aging of society, the number of lower limb fractures (including proximal femur fractures) has been increasing in Japan [1–3]. An increase in the number of proximal femur fractures is a major public health concern because they are one of the major causes of decline in activities of daily living (ADL), disability, dependency, and subsequent poor prognosis, including death [4–6].

Several epidemiological studies have investigated the incidence rates and risk factors of lower limb fractures in Japan [1–3, 7] as well as other countries around the world [5, 8] and demonstrated that older age, lower bone mineral density (BMD), a history of fracture, current smoking, alcohol intake, and lower calcium intake are major risk factors [8–13]. Some Japanese studies have investigated case-control studies in hospitals [4], and some have investigated the relationship between hip fractures and diabetes mellitus [14] or depression [15] or chronic obstructive pulmonary disease [16]. However, evidence from a nationwide population-based prospective study is scarce in Japan. Furthermore, a limited number of cohort studies have conducted detailed investigations on incidence rates and/or risk factors (including detailed dietary factors such as calcium intake) of lower limb fractures by gender and location of fracture, although risk factors of proximal femur fractures, which are highly related to osteoporosis, are different from those of fractures in other locations of the lower limbs [6, 7].

The present study aimed to investigate the incidence rates and risk factors (including dietary factors) of lower limb fractures by location in a long-term prospective cohort study of general Japanese men and women recruited from all regions of Japan.

## Materials and methods

### Study design, participants, and follow-up assessments

**Fig 1:**
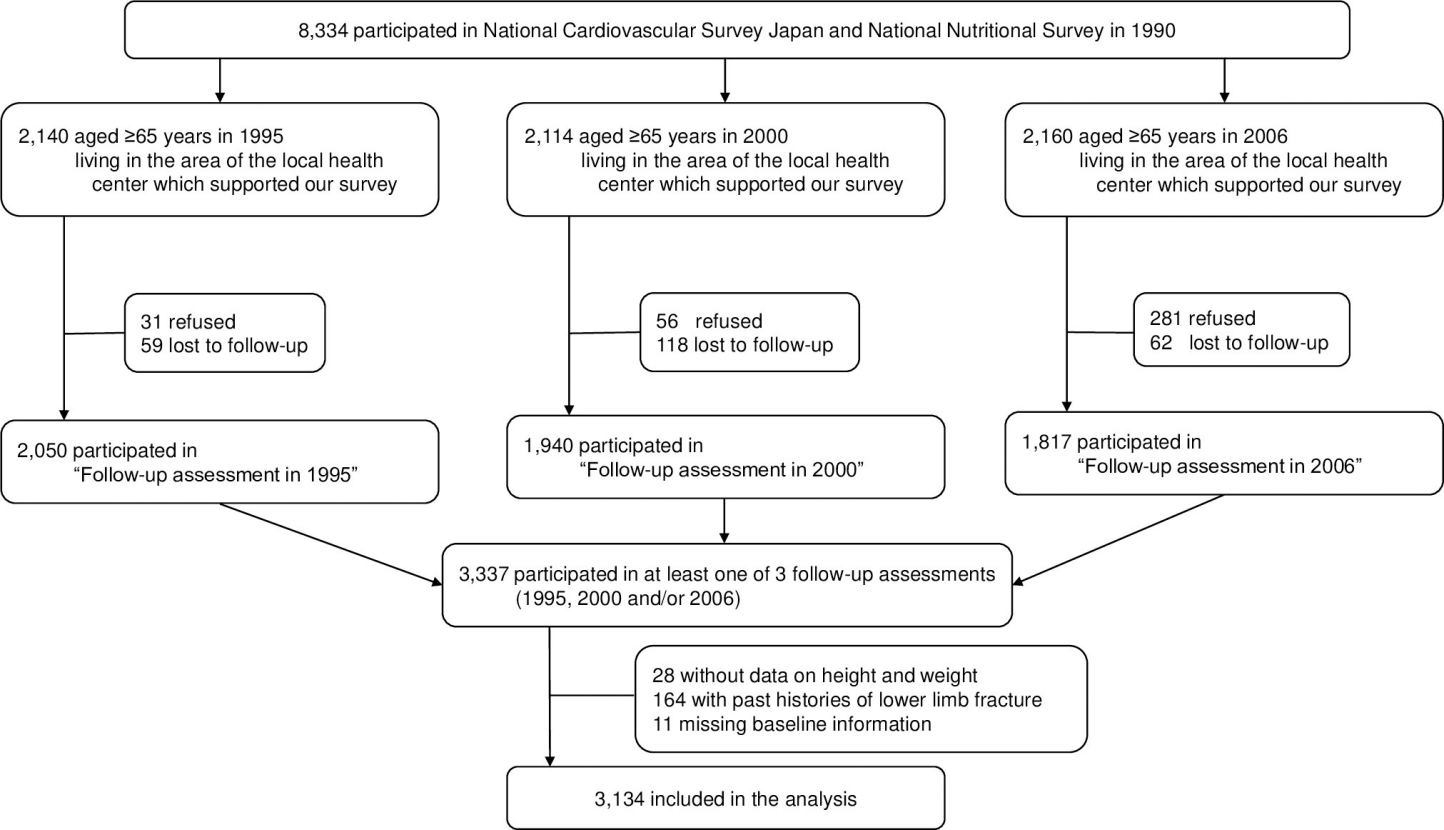
Flow diagram of participants.

NIPPON DATA 90 (National Integrated Project for Prospective Observation of Non-communicable Disease And its Trends in the Aged) is a large-scale, population-based cohort study of individuals who participated in the 4th National Cardiovascular Survey Japan [17] and the National Nutrition Survey (NNS) [18] in 1990. The details of this cohort study have been reported previously [19, 20]. In brief, a total of 8,384 residents (3,504 men and 4880 women, aged ≥30 years) from 300 randomly selected districts throughout Japan participated in the survey. The total population of residents aged ≥30 years in the selected districts was 10,956, and the participation rate was 76.5%. A flow diagram of the participants in the present analysis is shown in Fig 1.

In 1995, 2,140 elderly (≥65 years in 1995) participants, who lived in the area of the local health center that supported our survey, were invited to the “follow-up assessment 1995”. Among them, 31 refused, 59 relocated, and a total of 2,050 elderly participants (participation rate 95.8%) responded to the survey, including questionnaires on lower limb fractures, ADL, and instrumental ADL (IADL). In 2000, 2,114 elderly (≥65 years in 2000) participants with the same inclusion criteria were invited, and 1,940 (participation rate 91.8%) responded to the “follow-up assessment 2000”. In 2006, 2,114 elderly (≥65 years in 2000) participants with the same inclusion criteria were invited, and 1,817 (participation rate 81.1%) responded to the “follow-up assessment 2006”. Overall, 3,337 individuals participated in at least one of the three surveys (1995, 2000, and/or 2006). After excluding 203 participants (28 without data on height and weight, 164 with histories of lower limb fracture, 11 with missing information on time to lower limb fracture), 3,134 participants (1,827 women, 1,307 men, mean age 63.5 years) were included in the analysis.

### Ethics statement

In NIPPON DATA90, participants’ consent was obtained using the opt-out approach. Verbal consent was obtained for participants in the follow-up assessments in 1995, 2000, and 2006. The institutional review board of Shiga University of Medical Science approved the NIPPON DATA 90 (R2005-021).

### Outcomes

The primary outcome of the present analysis was the time to first lower limb fracture. We used home-visit interviews of the participants and their family members (if available) by public health nurses to assess the outcome at each follow-up assessment (1995, 2000, and 2006); if this was impractical, the questions were asked over the phone or questionnaires were mailed. At each follow-up assessment, detailed information on the history of lower limb fractures, including location (proximal femur fractures, knee, ankle, toes, etc.) and onset date, was obtained. Only the first lower limb fracture in each participant was included in the analysis.

### Baseline examinations

The region of residence was defined as Eastern Japan (Hokkaido, Tohoku, Kanto, and Chubu regions) and Western Japan (Kansai, Chugoku, Shikoku, and Kyushu/Okinawa regions). Body mass index (BMI) was calculated as weight divided by height squared (kg/m^2^). Non-fasting blood samples were collected, and serum albumin levels were analyzed using an auto-analyzer (SMA12/60; Technicon, Tarrytown, New York, USA). Smoking status was classified as non-smoker (never or ex-smoker) or current smoker. Alcohol use was categorized into non-drinkers (never or former drinkers) or current alcohol drinkers. Regular physical activity was defined as no exercise (individuals with no regular physical activity) or current exercise (individuals regularly involved in physical activity). We also used the data of the NNS conducted in 1990 [18]. The nutritional survey was conducted by staff at the regional health centers under the supervision of the Health Promotion and Nutrition Division. Detailed methods of dietary assessment, estimation of individual intake of nutrients, and food groups have been described elsewhere [19, 20]. In brief, a food intake survey using weighed food records on three consecutive representative days (excluding weekends, national holidays, and ceremonial occasions) was conducted in mid-November 1990. The weight of each food item (e.g., carrot, spinach, capsicum, tomato, beef, pork, chicken, rice, soy sauce, salt, sugar, etc.) used for breakfast, lunch, and dinner and that of the leftover was recorded using a weight meter by a member of each household. During the survey, trained dietitians visited each household at least once a day to check their records. The quantity of food intake was calculated from the recorded dietary intake status and then the quantity of nutrient intake was calculated from the quantity of food intake based on food composition tables (Standard Tables of Food Composition in Japan [4th edition]) [21] and the composition tables of restaurant, prepared, and processed food for the NNS in Japan [19, 20]. These food composition tables listed the energy and nutrient contents per 100 g of each food. Food and nutrient intake of each household member was estimated by dividing the household intake data of NNS1990 proportionally using the average intake by sex and age groups calculated for NNS conducted in 1995 [19, 20]. Food items were classified into eight food groups ([1] meats, [2] fish and shellfish, [3] vegetables, [4] milk and dairy products, [5] fruits, [6] rice, [8] beans, [7] mushrooms), and intake was calculated as grams/1,000 kcal among each group. For energy supply of nutrients, the intake was calculated as the percentage of total energy intake (%kcal). Other nutrients were calculated relative to the total dietary intake (grams/1,000 kcal).

### Statistical analysis

Because incidence rates and risk factors of lower limb fractures were shown to be heterogeneous between men and women, incidence rats and risk factors were investigated according to sex [7]. The incidence rates were estimated using the person-year approach. The effects of risk factors (age, region, BMI, smoking status, alcohol intake, physical activity, and dietary factors) on outcomes were assessed using Cox’s proportional hazards models separately for men and women. Multivariable analyses included the following variables: Model 1, age and BMI; Model 2, age, height, and weight; Model 3, Model 1 + smoking status, alcohol intake, physical activity, and serum albumin; Model 4, Model 1 + smoking status, alcohol intake, physical activity, total energy, protein, and calcium intake; and Model 5, Model 1 + smoking status, alcohol intake, physical activity, total energy, and food groups. Since age and BMI did not show linear associations with outcomes, they were considered categorical variables. For other risk factors of continuous variables, hazard ratios (HRs) were calculated using a 1 standard deviation (SD) higher value. The hypothesis testing was two-sided, with a significance level of 0.05. SPSS version 22 (IBM Corporation, Chicago, IL, USA) was used for all analyses.

## Results

The baseline characteristics of study participants are shown in Table 1.

**Table 1:** Baseline characteristics of participants: NIPPON DATA90, 1,827 women and 1,307 men in 1990.

	Total(n = 3134)		Women (n = 1827)		Men (n = 1307)		*P*-value
Age (years)	63.5	± 8.5	63.8	±8.8	63.1	± 8.1	0.020
Region of residence							
Eastern Japan (%)	59.6		58.7		60.9		
Western Japan (%)	40.4		41.3		39.1		
Height (cm)	153.6	± 8.8	148.3	± 6.1	161.0	± 6.1	<0.001
Weight (kg)	54.7	± 9.7	51.4	± 8.6	59.4	± 9.2	<0.001
Body mass index (kg/m^2^)	23.1	± 3.2	23.3	± 3.4	22.9	± 3.1	<0.001
Serum albumin (g/dl)	4.4	± 0.3	4.4	± 0.3	4.4	± 0.3	0.073
Current smoking (%)	23.5		6.4		47.5		<0.001
Current drinking (%)	26.0		3.8		57.1		<0.001
Current exercise (%)	24.1		21.8		27.2		0.001
Dietary intake							
Total energy (kcal)	2008	± 476	1819	± 381	2273	± 468	<0.001
Protein (%kcal)	16.0	± 2.0	16.1	± 2.0	15.8	± 1.9	<0.001
Calcium (mg/1000kcal)	275.9	± 84.9	307.7	± 90.9	261.2	± 79.8	0.418
Meats (g/1000kcal)	28.8	± 16.1	28.7	± 16.2	29.0	± 15.9	0.601
Fish and shellfish (g/1000kcal)	52.3	± 22.7	52.0	± 23.0	52.9	± 22.3	0.232
Vegetable (g/1000kcal)	131.7	± 47.8	132.5	± 48.7	130.7	± 46.4	0.292
Milk and dairy products (g/1000kca)	57.3	± 45.3	58.0	± 45.9	56.4	± 44.4	0.340
Fruits (g/1000kcal)	72.2	± 49.4	73.2	± 49.4	70.3	± 49.3	0.108
Cereals (g/1000kcal)	143.9	± 28.2	143.6	± 28.3	144.4	± 28.0	0.445
Rice (g/1000kcal)	103.5	± 30.6	103.4	± 30.8	103.6	± 30.4	0.868
Soybean (g/1000kcal)	38.3	± 22.0	38.3	± 22.5	38.4	± 21.3	0.937
Mushrooms (g/1000kcal)	5.9	± 6.9	5.9	± 7.1	5.9	± 6.6	0.885

In women, the mean age was 63.8 (SD 8.8) years, mean BMI was 23.3 (SD 3.4) kg/m^2^, and mean total energy intake was 1819 (SD 381) kcal. In men, the mean age was 63.1 (SD 8.1) years, mean BMI was 22.9 (SD 3.1) kg/m^2^, and mean total energy intake was 2273 (SD 468) kcal. Statistical differences were observed between men and women in total energy intake, smoking, and alcohol drinking status (all P<0.05).

During the mean follow-up period of 12.1 years, 281 lower limb fractures (109 proximal femur fractures and 172 others [including 42 patella and 33 ankle fractures]) were observed. The incidence rates of proximal femur fracture were 2.8 in women and 1.3 in men, those of other lower limb fracture were 4.6 in women and 2.3 in men, and those of total lower limb fracture were 7.6 in women and 3.7 in men. Incidence rates of lower limb fractures are shown by baseline age and sex (Table 2).

**Table 2:** Incident rates (per 1000 person-years) of first-ever lower limb fracture by baseline age and sex, NIPPON DATA90, 1,827 women and 1,307 men.

	Proximal femur fracture		Other lower limb fracture		Total lower limb fracture	
Baseline age	Women	Men	Women	Men	Women	Men
<60 years	0.6(0.1 to 1.1)	0.7(0.1 to 1.1)	3.8(2.5 to 5.1)	1.4(0.5 to 2.3)	4.5(3.1 to 5.9)	2.2(1.1 to 3.3)
60–69 years	2.8(2.1 to 3.5)	1.3(0.4 to 2.2)	4.9(3.4 to 6.4)	3.5(2 to 5)	7.7(5.8 to 9.6)	4.7(3 to 6.4)
≥70 years	7.2(4.7 to 9.7)	2.9(0.8 to 5)	4.8(2.8 to 6.8)	1.6(0 to 3.2)	12.1(8.8 to 15.4)	4.6(1.9 to 7.3)
All age	2.8(2.1 to 3.5)	1.3(0.7 to 1.9)	4.6(3.7 to 5.5)	2.3(1.5 to 3.1)	7.6(6.4 to 8.8)	3.7(2.7 to 4.7)

Proximal femur fracture: P<0.001 for 60–69 years versus <60 years and P<0.001 for ≥70 years versus <60 years in women; P = 0.22 for 60–69 years versus <60 years and P = 0.008 for ≥70 years versus <60 years in men

Other lower limb fracture: P = 0.145 for 60–69 years versus <60 years and P = 0.161 for ≥70 years versus <60 years in women; P = 0.017 for 60–69 years versus <60 years and P = 0.721 for ≥70 years versus <60 years in men

Total lower limb fracture: P = 0.003 for 60–69 years versus <60 years and P<0.001 for ≥70 years versus <60 years in women; P = 0.009 for 60–69 years versus <60 years and P = 0.028 or ≥70 years versus <60 years in men

The incidence rates (per 1000 person-years) of proximal femur fractures was higher with older age in both women (0.6 for <60 years, 3.0 for 60–69 years, and 7.0 for ≥70 years) and men (0.7 for <60 years, 1.3 for 60–69 years, and 2.9 for ≥70 years). However, there were no clear associations between age and other lower limb fractures. When the proximal femur and other fractures were combined, a linear association between age and total lower limb fractures was observed only in women. Multivariable-adjusted HRs and 95% confidence intervals (95% CIs) for proximal femur fractures in women are shown in Table 3.

**Table 3:** Multivariable-adjusted hazard ratios and 95% confidence intervals for proximal femur fractures: NIPPON DATA90, 1,827 women.

	Hazard ratio (95% confidence interval)									
	Model 1		Model 2		Model 3		Model 4		Model 5	
Age (vs 49–59 years)										
60–69 years	5.07	(2.07–12.40) ***	5.41	(2.20–13.35) ***	6.10	(2.31–16.07) ***	5.05	(2.04–12.47) ***	5.10	(2.05–12.68) ***
≥70 years	12.24	(5.05–29.67) ***	14.26	(5.64–36.02) ***	14.63	(5.51–38.83) ***	11.65	(4.65–29.18) ***	11.39	(4.55–28.53) ***
Region of residence	1.19	(0.72–1.97)	1.18	(0.72–1.95)	1.06	(0.63–1.79)	1.21	(0.73–1.99)	1.09	(0.65–1.83)
(Western vs Eastern Japan)										
Height (per1SD increase)			1.51	(1.11–2.02) **						
Weight (per1SD increase)			0.57	(0.41–0.79) ***						
BMI (kg/m^2^)										
<20	2.23	(1.10–4.46) *			2.47	(1.20–5.07) *	2.19	(1.08–4.41) *	2.27	(1.12–4.60) *
≥20–22.4	1.47	(0.73–2.92)			1.41	(0.68–2.91)	1.51	(0.76–3.02)	1.56	(0.78–3.13)
≥22.5–24.9	1	(Reference)			1	(Reference)	1	(Reference)	1	(Reference)
≥25	0.73	(0.33–1.60)			0.64	(0.27–1.51)	0.73	(0.33–1.62)	0.74	(0.33–1.63)
Current smoking					0.27	(0.04–1.93)	0.24	(0.03–1.77)	0.23	(0.03–1.64)
Current regular exercise					1.75	(0.85–3.57)	1.69	(0.85–3.33)	1.67	(0.84–3.33)
Serum albumin					0.93	(0.67–1.29)				
Total energy intake^†^							0.90	(0.68–1.20)	0.89	(0.67–1.18)
Protein intake^†^							0.88	(0.66–1.18)		
Calcium intake^†^							1.06	(0.81–1.39)		
Meats intake^†^									1.01	(0.77–1.31)
Fish and shellfish intake^†^									1.04	(0.80–1.35)
Vegetable intake^†^									0.63	(0.47–0.86) *
Milk and dairy products intake^†^									0.93	(0.71–1.21)
Fruits intake^†^									1.09	(0.86–1.39)
Cereals^†^									1.06	(0.80–1.41)
Rice^†^									0.96	(0.73–1.26)
Soybean^†^									1.01	(0.80–1.29)
Mushrooms^†^									0.89	(0.65–1.22)

*P<0.05

**P<0.01

***P<0.001

^†^per 1standard deviation (6.1cm for height, 8.6kg for weight, 3.4kg/m^2^ for body mass index, 0.3g/dl for serum albumin, 381kcal for total energy, 2.0%kcal protein, 90.9mg/1000kcal for calcium, 16.2g/1000kcal for meats, 23.0g/1000kcal for fish, 48.7g/1000kcal for vegetable, 45.9g/1000kcal for milk, 49.4g/1000kcal for fruits, 28.3g/1000kcal for cereals, 30.8g/1000kcal for rice,22.5g/1000kcal for soybean,7.1 g/1000kcal for mushrooms) increase.

Abbreviations: HR, hazard ration; 95% Cl, 95% confidence interval; BMI, body mass index.

Age was significantly associated with an increased risk of proximal femur fractures in all models (Models 1 to 5). Participants with low BMI (<20 kg/m^2^) had significantly higher risks of proximal femur fractures than the reference group (BMI, 22.5 to 24.9 kg/m^2^) in Models 1, 3, 4, and 5. Furthermore, vegetable intake was associated with lower risks of proximal femur fractures in Model 5 (HR, 0.63; 95% CI 0.47–0.86). Calcium intake was not clearly associated with the risk of proximal femur fractures in Model 4. Among men, there were no significant predictors of proximal femur fractures, except for age, in Models 1 to 3 (Table 4).

**Table 4:** Multivariable-adjusted hazard ratios and 95% confidence intervals for proximal femur fractures: NIPPON DATA90, 1,307 men.

	Hazard ratio (95% confidence interval)									
	Model 1		Model 2		Model 3		Model 4		Model 5	
Age (vs 49–59 years)										
60–69 years	2.11	(0.68–6.54)	2.34	(0.75–7.28)	2.07	(0.65–6.56)	1.56	(0.48–5.04)	1.56	(0.48–5.08)
≥70 years	4.98	(1.52–16.35) **	6.56	(1.92–22.43) **	5.06	(1.42–17.94) *	2.87	(0.75–10.93)	3.33	(0.86–12.97)
Region of residence	0.71	(0.27–1.85)	0.71	(0.27–1.87)	0.76	(0.29–1.98)	0.75	(0.29–1.97)	0.64	(0.24–1.72)
(Western vs Eastern Japan)										
Height (per1SD increase)			1.40	(0.83–2.33)						
Weight (per1SD increase)			1.10	(0.69–1.76)						
BMI (kg/m^2^)										
<20	2.30	(0.51–10.31)			2.22	(0.49–10.11)	1.95	(0.43–8.87)	2.05	(0.45–9.33)
≥20–22.4	2.29	(0.57–9.14)			2.21	(0.55–8.87)	2.09	(0.52–8.43)	2.34	(0.56–9.72)
≥22.5–24.9	1	(Reference)			1	(Reference)	1	(Reference)	1	(Reference)
≥25	3.54	(0.91–13.69)			3.53	(0.91–13.72)	3.52	(0.91–13.66)	3.73	(0.93–14.93)
Current smoking					1.59	(0.65–3.90)	1.51	(0.62–3.71)	1.65	(0.66–4.15)
Current alcohol intake					0.94	(0.38–2.31)	0.99	(0.37–2.64)	1.06	(0.42–2.62)
Current regular exercise					1.03	(0.37–2.56)	1.01	(0.38–2.70)	0.99	(0.36–2.63)
Serum albumin					0.98	(0.56–1.73)				
Total energy intake^†^							0.57	(0.33–0.98) *	0.61	(0.35–1.05)
Protein intake^†^							0.82	(0.50–1.35)		
Calcium intake^†^							1.15	(0.73–1.82)		
Meats intake^†^									0.89	(0.53–1.49)
Fish and shellfish intake^†^									0.96	(0.61–1.50)
Vegetable intake^†^									0.90	(0.56–1.46)
Milk and dairy products intake^†^									1.44	(1.00–2.05)
Fruits intake^†^									0.86	(0.53–1.40)
Cereals^†^									1.13	(0.70–1.82)
Rice^†^									0.86	(0.53–1.42)
Soybean^†^									1.42	(1.00–2.02)
Mushrooms^†^									1.25	(0.85–1.84)

*P<0.05

**P<0.01

***P<0.001

^†^per 1standard deviation (6.1cm for height, 9.2kg for weight, 3.1kg/m^2^ for body mass index, 0.3g/dl for serum albumin, 468kcal for total energy, 1.9%kcal protein, 79.8mg/1000kcal for calcium, 15.9g/1000kcal for meats, 22.3g/1000kcal for fish, 46.4g/1000kcal for vegetable, 44.4g/1000kcal for milk, 49.3g/1000kcal for fruits, 28.0g/1000kcal for cereals, 30.4g/1000kcal for rice,21.3g/1000kcal for soybean,6.6 g/1000kcal for mushrooms) increase.

Abbreviations: HR, hazard ration; 95% Cl, 95% confidence interval; BMI, body mass index.

Multivariable-adjusted HRs and 95% CIs for total lower limb fractures are shown in Table 5 (women) and Table 6 (men).

**Table 5:** Multivariable-adjusted hazard ratios and 95% confidence intervals for total lower limb fractures: NIPPON DATA90, 1,827 women.

	Hazard ratio (95% confidence interval)									
	Model 1		Model 2		Model 3		Model 4		Model 5	
Age (vs 49–59 years)										
60–69 years	1.84	(1.23–2.73) **	1.86	(1.23–2.78) **	*2*.*00*	(1.33–3.01) **	1.87	(1.25–2.81) ***	1.93	(1.28–2.92) ***
≥70 years	3.04	(2.00–4.62) ***	3.10	(1.96–4.90) ***	*3*.*19*	(2.03–5.00) ***	3.15	(2.02–4.92) ***	3.20	(2.04–5.01) ***
Region of residence	1.35	(0.99–1.86)	1.35	(0.99–1.86)	1.30	(0.93–1.80)	1.38	(1.01–1.90)	1.31	(0.95–1.81)
(Western vs Eastern Japan)										
Height (per1SD increase)			1.04	(0.85–1.26)						
Weight (per1SD increase)			0.95	(0.79–1.15)						
BMI (kg/m^2^)										
<20	1.36	(0.84–2.20)			1.44	(0.88–2.34)	1.35	(0.83–2.18)	1.37	(0.85–2.22)
≥20–22.4	1.16	(0.75–1.79)			1.12	(0.71–1.75)	1.16	(0.75–1.80)	1.17	(0.75–1.82)
≥22.5–24.9	1	(Reference)			1	(Reference)	1	(Reference)	1	(Reference)
≥25	1.09	(0.71–1.68)			0.98	(0.62–1.53)	1.09	(0.71–1.68)	1.10	(0.72–1.70)
Current smoking					0.61	(0.27–1.41)	0.60	(0.26–1.36)	0.57	(0.25–1.30)
Current alcohol intake					1.40	(0.60–3.24)	1.34	(0.58–3.09)	1.31	(0.56–3.04)
Current regular exercise					1.72	(0.86–2.78)	1.67	(1.08–2.63) *	1.67	(1.06–2.63) *
Serum albumin					0.95	(0.77–1.17)				
Total energy intake^†^							0.98	(0.83–1.16)	0.98	(0.83–1.16)
Protein intake^†^							0.94	(0.78–1.13)		
Calcium intake^†^							1.10	(0.92–1.31)		
Meats intake^†^									1.03	(0.87–1.21)
Fish and shellfish intake^†^									1.03	(0.87–1.23)
Vegetable intake^†^									0.85	(0.72–1.01)
Milk and dairy products intake^†^									0.98	(0.83–1.16)
Fruits intake^†^									1.04	(0.87–1.23)
Cereals^†^									1.03	(0.87–1.23)
Rice^†^									0.99	(0.84–1.17)
Soybean^†^									1.11	(0.96–1.28)
Mushrooms^†^									1.07	(0.90–1.27)

*P<0.05

**P<0.01

***P<0.001

^†^per 1standard deviation (6.1cm for height, 8.6kg for weight, 3.4kg/m^2^ for body mass index, 0.3g/dl for serum albumin, 381kcal for total energy, 2.0%kcal protein, 90.9mg/1000kcal for calcium, 16.2g/1000kcal for meats, 23.0g/1000kcal for fish, 48.7g/1000kcal for vegetable, 45.9g/1000kcal for milk, 49.4g/1000kcal for fruits, 28.3g/1000kcal for cereals, 30.8g/1000kcal for rice,22.5g/1000kcal for soybean,7.1 g/1000kcal for mushrooms) increase.

Abbreviations: HR, hazard ration; 95% Cl, 95% confidence interval; BMI, body mass index.

**Table 6:** Multivariable-adjusted hazard ratios and 95% confidence intervals for total lower limb fractures: NIPPON DATA90, 1,307 men.

	Hazard ratio (95% confidence interval)									
	Model 1		Model 2		Model 3		Model 4		Model 5	
Age (vs 49–59 years)										
60–69 years	2.43	(1.29–4.58) **	2.43	(1.28–4.61) **	2.06	(1.06–4.01) **	2.21	(1.14–4.28) *	2.28	(1.18–4.40) *
≥70 years	2.54	(1.14–5.63) *	2.58	(1.13–5.88) *	2.43	(1.03–5.57) *	2.29	(0.95–5.53)	2.54	(1.06–6.09) *
Region of residence	0.73	(0.41–1.29)	0.72	(0.41–1.28)	0.64	(0.34–1.18)	0.77	(0.43–1.37)	0.73	(0.41–1.32)
(Western vs Eastern Japan)										
Height (per1SD increase)			0.99	(0.72–1.36)						
Weight (per1SD increase)			1.11	(0.83–1.50)						
BMI (kg/m^2^)										
<20	0.77	(0.32–1.89)			0.73	(0.30–1.78)	0.67	(0.27–1.65)	0.70	(0.29–1.73)
≥20–22.4	1.29	(0.66–2.54)			1.11	(0.55–2.22)	1.20	(0.61–2.38)	1.26	(0.63–2.50)
≥22.5–24.9	1	(Reference)			1	(Reference)	1	(Reference)	1	(Reference)
≥25	1.24	(0.59–2.57)			1.15	(0.54–2.45)	1.21	(0.58–2.53)	1.23	(0.59–2.58)
Current smoking					1.33	(0.76–2.33)	1.43	(0.83–2.48)	1.41	(0.81–2.45)
Current alcohol intake					1.11	(0.63–1.98)	1.16	(0.66–2.03)	1.23	(0.70–2.17)
Current regular exercise					1.47	(0.88–2.86)	1.59	(0.81–3.13)	1.56	(0.80–3.13)
Serum albumin					0.80	(0.56–1.13)				
Total energy intake^†^							0.79	(0.57–1.08)	0.84	(0.62–1.16)
Protein intake^†^							0.82	(0.60–1.12)		
Calcium intake^†^							1.01	(0.74–1.38)		
Meats intake^†^									0.89	(0.66–1.20)
Fish and shellfish intake^†^									0.87	(0.65–1.15)
Vegetable intake^†^									1.18	(0.92–1.53)
Milk and dairy products intake^†^									1.09	(0.83–1.44)
Fruits intake^†^									0.76	(0.54–1.06)
Cereals^†^									1.08	(0.81–1.46)
Rice^†^									0.88	(0.66–1.18)
Soybean^†^									1.15	(0.91–1.45)
Mushrooms^†^									1.07	(0.82–1.40)

*P<0.05

**P<0.01

***P<0.001

^†^per 1standard deviation (6.1cm for height, 9.2kg for weight, 3.1kg/m^2^ for body mass index, 0.3g/dl for serum albumin, 468kcal for total energy, 1.9%kcal protein, 79.8mg/1000kcal for calcium, 15.9g/1000kcal for meats, 22.3g/1000kcal for fish, 46.4g/1000kcal for vegetable, 44.4g/1000kcal for milk, 49.3g/1000kcal for fruits, 28.0g/1000kcal for cereals, 30.4g/1000kcal for rice,21.3g/1000kcal for soybean,6.6 g/1000kcal for mushrooms) increase.

Abbreviations: HR, hazard ration; 95% Cl, 95% confidence interval; BMI, body mass index.

Age was significantly associated with increased risks of total lower limb fractures in women and men (all models). In women, regular exercise was also associated with an increased risk of total lower limb fractures (Models 4 and 5). Furthermore, vegetable intake was not significantly associated with total lower limb fractures in women in Model 5; however, relevant trends were also observed. Calcium intake was not clearly associated with the risk of total lower limb fractures in Model 4. Among men, there were no significant predictors of total lower limb fractures, except for age. In men, milk intake was associated with lower proximal femur fractures in Model 5.

## Discussion

In the present long-term cohort study of a representative general Japanese population, aging was a significant risk factor for proximal femur and total lower limb fractures in both men and women. With regard to modifiable risk factors, low BMI and low vegetable intake were associated with increased risks of proximal femur fractures among women. Furthermore, less intake of vegetables and regular exercise were associated with an increased risk of total lower limb fractures. In contrast, there were no significant modifiable risk factors for proximal femur or total lower limb fractures in men. Calcium intake was not a significant predictor of total lower limb fractures in both men and women.

The following content has been added to the discussion. Since no other studies of local residents throughout Japan were found, the incidence of peri-femoral fractures in men and women could not be compared with that reported in other studies. However, we were able to determine the incidence of proximal femur fractures diagnosed by an orthopedic surgeon nationwide or in certain areas in this study. Similarly, incidence increased with age; the incidence was high in all age groups in this study. However, some patients diagnosed by orthopedics may be admitted to a long-term care health facility. It is speculated that the difference in activity level is related to the difference in the incidence of proximal femur fractures between this study and other studies.

In the present analysis from NIPPON DATA 90, BMI <20 kg/m^2^ was associated with increased risks of proximal femur fractures in women. Comparable findings were observed in a previous observational study [15]. The mechanisms underlying the association between low BMI and proximal femur fractures are not clear; however, extreme thinness may be associated with lower levels of BMD, muscle volume, and/or subcutaneous adipose tissue of the hip, which are likely to be associated with increased risks of falls and decreased protection against shock toward the femur [22, 23]. These mechanisms may also explain the lack of clear associations between BMI and fractures in other locations of the lower limbs, such as the knees and toes, where the bones are directly exposed to external shocks. In men, a higher BMI tended to be associated with a higher risk of proximal femur fracture. This sex difference may be due to differences in fat distribution by sex [24].

In the present analysis, the risks of proximal femur fractures and any lower limb fractures were significantly lower in women with a higher intake of vegetables at baseline. Similar findings were reported in a few case-control studies that reported a link between vegetable intake and lower risks of forearm fractures as well as proximal femur fractures [25]. A systematic review concluded that there is still insufficient evidence to support the protective effects of vegetables against fractures [26]. The findings in the present study may partly be explained by the increased intake of vitamin K from certain types of vegetables, which enhances bone formation and increases the strength of the bone through γ-carboxylation of osteocalcin [27]. Increased intake of potassium, magnesium, and boron from vegetables has also been shown to move the body into an alkaline environment where osteoblast function and bone formation are enhanced and osteoclast activity and bone loss are increased [28].

Several observational studies have reported the protective effects of regular physical activity on the prevention of lower limb fractures [29, 30]. In contrast, in the present analysis, regular physical activity was associated with increased risks of total lower limb fractures in women. Although excessive exercise may be a trigger for falls and subsequent fractures, further investigations are required to clarify the association between physical activity and lower limb fractures.

Previous observational studies demonstrated the protective effects of dietary calcium intake and fractures [9, 31], while randomized controlled trials of increased intake of dietary calcium or supplementary intake of calcium did not provide significant protection against fractures [32, 33]. The present long-term prospective study of the general Japanese population used detailed data on nutritional intake from the NNS but did not find any significant associations between calcium intake and fractures (proximal femur fractures and any lower limb fractures) among men and women. Inconsistent findings may be attributable to differences in study design, characteristics of participants, definition of outcomes, etc.

In the present analysis, incidence rates (per 1000 person-years) of proximal femur fractures were 2.8 in women and 1.3 in men. In a hospital-based study conducted in the Niigata Prefecture, Japan, incidence rates were comparable with those in the present analysis (3.1 in women and 0.9 in men) [34]. In contrast, a nationwide hospital-based study in Japan reported lower incidence rates (2.1 among women and 0.6 among men) [3]. Discrepancies in findings may be attributable to differences in study design, setting, participants, definition of outcomes, etc.

The strengths of this study include the study design (a long-term cohort study), generalizability of the findings (nationwide population-based study of Japanese), and detailed analysis of risk factors (including detailed dietary factors) of lower limb fractures by sex and fracture location. This study has several limitations. First, outcomes were defined based on information obtained from interviews with participants or their family members and were not confirmed by medical charts or imaging. Second, more detailed clinical information (e.g., location of proximal femur fractures) was not available in the present study. Although we attempted to avoid memory bias by home-visit interviews of the participants and their family members (if available) three times during the follow-up period, the outcome might have been underreported in some participants. Third, information on some important risk factors of lower limb fractures (e.g., presence and/or treatment of osteoporosis, history of rheumatoid arthritis, and use of steroids) was not available in this study. Finally, we used only baseline diet factors, while food intake may change over time. This fact might have resulted in an underestimation of the association between diet factors and lower limb fractures.

## Conclusions

Aging was a significant risk factor for proximal femur and total lower limb fractures in both men and women. With regard to modifiable risk factors, low BMI, low intake of vegetables, and regular exercise were associated with increased risks of proximal femur and/or total lower limb fractures in a population-based long-term prospective cohort study of general Japanese women. Population strategies to reduce extreme thinness and increase the intake of vegetables among women appears to provide protection against the increasing burden of lower limb fractures in Japan.
